# Improvements of Developed Graphite Based Composite Anti-Aging Agent on Thermal Aging Properties of Asphalt

**DOI:** 10.3390/ma13184005

**Published:** 2020-09-10

**Authors:** Zhihui Hu, Tao Xu, Pengfei Liu, Markus Oeser, Haopeng Wang

**Affiliations:** 1 College of Civil Engineering, Nanjing Forestry University, 159 Longpan Road, Nanjing 210037, China; hzh@njfu.edu.cn; 2 Institute of Highway Engineering, Rheinisch-Westfälische Technische Hochschule Aachen University, Mies-van-der-Rohe-Street 1, 52074 Aachen, Germany; liu@isac.rwth-aachen.de (P.L.); oeser@isac.rwth-aachen.de (M.O.); 3 Section of Pavement Engineering, Faculty of Civil Engineering and Geosciences, Delft University of Technology, Stevinweg 1, 2628 CN Delft, The Netherlands

**Keywords:** asphalt, thermal-oxidative aging, composite anti-aging agent, volatile constituents, synergistic effect

## Abstract

To reduce the thermal-oxidative aging of asphalt and the release amount of harmful volatiles during the construction of asphalt pavement, a new composite anti-aging agent was developed. Since the volatiles were mainly released from saturates and aromatics during the thermal-oxidative aging of asphalt, expanded graphite (EG) was selected as a stabilizing agent to load magnesium hydroxide (MH) and calcium carbonate (CaCO_3_) nanoparticles for preparing the anti-aging agents of saturates and aromatics, respectively. Thermal stability and volatile constituents released from saturates and aromatics before and after the thermal-oxidative aging were characterized using the isothermal Thermogravimetry/Differential Scanning Calorimetry-Fourier Transform Infrared Spectrometer test (TG/DSC-FTIR test). Test results indicate that anti-aging agents of EG/MH and EG/CaCO_3_ effectively inhibit the volatilization of light components in asphalt and improve the thermal stability of saturates and aromatics. Then, the proportions of EG, MH, and CaCO_3_ added in the developed composite anti-aging agent of EG/MH/CaCO_3_ are 2:1:3 by weight. EG/MH/CaCO_3_ plays a synergetic effect on inhibiting the thermal-oxidative aging of asphalt, and reduces the release amount of harmful volatiles during the thermal-oxidative aging after EG/MH/CaCO_3_ is added into asphalt at the proposed content of 10 wt.%. EG plays a synergistic role with MH and CaCO_3_ nanoparticles to prevent the chain reactions, inhibiting the thermal-oxidative aging of asphalt.

## 1. Introduction

Asphalt is a mixture of hydrocarbon and non-hydrocarbon compounds with very complex components. Asphalt is one of the most widely used binder materials of pavement engineering in China. However, it is vulnerable to aging during the construction and service life of asphalt pavement. When exposed to heat, sunlight, oxygen, and moisture, asphalt becomes harder, leading to a series of pavement distresses such as crack, raveling, pot holes, etc. [1]. Moreover, the released volatile organic compounds are harmful to the natural environment and construction workers’ health when the thermal-oxidative reaction of asphalt occurs at high temperature [2]. Thermal-oxidative aging exists in the construction and service processes of asphalt pavement, including mixing, paving, rolling, and service stage [3,4]. Thus, some efficient anti-aging agents are developed to prolong the service life of asphalt pavement. 

Some efforts have been also made to improve the aging resistance of asphalt by adding various modifiers. The development of anti-aging agent is generally considered from the standpoint of antioxidants, light stabilizers, and heat stabilizers [5,6]. Carbon black, montmorillonite, antioxidant, and ultraviolet absorber (UVA), etc. are usually used as additives to inhibit the thermal- and photo- oxidative aging of asphalt [6]. A multi-dimensional nanomaterial composed of layered inorganic silicates has been used to synergistically improve thermal- and photo-oxidative aging resistance [7,8]. Zare-Shahabadi et al. [9] found that the addition of nanoclay increased the viscosity of asphalt, and improved the anti-aging, anti-rutting, and anti-fatigue properties of asphalt. 

Recently, EG has been paid more attention because it is a new type of mesoporous carbon material and has higher heat transfer and adsorption properties [10]. When compared with other adsorbents, EG shows a stronger ability to adsorb and fix oils due to its porous structures, as well as excellent heat and mass transfer performances [11,12,13]. As a result, EG can be better dispersed in the aromatics, saturates, and asphalt. In addition, EG is easy to adsorb oil and organic small molecules, which is suitable to use as the carrier of anti-aging agents of saturates and aromatics [11]. 

Additionally, it is known that at least one dimension of nanomaterial is in a nanoscale range of 1–100 nm. The ratio of the number of atoms to the total number of atoms on the surface of nano crystalline particles increases sharply with the decrease in particle size, which further causes the changes of material properties [14]. In recent years, more inorganic nanomaterials have been used as asphalt modifiers [14,15]. MH and CaCO_3_ nanoparticles were often used to improve thermal stability of polymer materials [16,17,18].

The MH nanoparticle, as a kind of high efficient flame retardant, has strong thermal stability and smoke suppression property [19,20]. MH nanoparticles show the volume effect and quantum size effect after nano crystallization, which improves the compatibility between MH and asphalt. MH also has a certain adsorption effect [21,22,23]. Additionally, MH is alkaline, which can not only play a role of filling, adsorption, and improve the thermal stability, but also neutralize some acid groups or gaseous products, such as –COOH, SO_2_, etc. [24]. 

Wu et al. [25] added MH into natural fiber-reinforced composites, and found that MH significantly enhanced the compatibility between fibers and polymer matrix, improving the moisture-damage resistance and mechanical properties of composites, and retarding aging process. Zhu et al. [26] used MH to modify a microporous polymer nanotube for improving its thermal stability, and reported that the modified microporous polymer nanotubes had the great potential application as a promising thermal insulation coating material for thermal energy saving.

Nano CaCO_3_ has a higher thermal stability, spatial stereoscopic structure, so that it has better dispersity in polymeric materials [24,26,27,28]. According to previous reports, the addition of CaCO_3_ nanoparticles could effectively improve the thermal stability and mechanical properties of polymer [29]. Moreover, the surface of active nano CaCO_3_ was oleophilic and hydrophobic, and it had a good compatibility with oil components, which could effectively improve or adjust the rheological properties of asphalt [24].

Nazari et al. [14] found that the anti-aging performance of asphalt was improved by adding CaCO_3_ nanoparticles, and the CaCO_3_ content affected fatigue property of modified asphalt. Xing et al. [30] proved that CaCO_3_ nanoparticles improved the high temperature stability and anti-rutting ability of asphalt by different reinforcement mechanisms. Zhai et al. [18] pointed out that asphalt modified by 5 wt.% nano CaCO_3_ and 4 wt.% SBR modifier had superior anti-rutting and creep characteristics at high temperature when compared with the SBS modified asphalt. 

Currently, most developed anti-aging agents of asphalt aimed at ultraviolet aging or long-term aging in natural environment, but ignored the adverse effect of short-term thermal-oxidative aging on the durability of asphalt pavement and the workers’ health at the construction stage. Further, although there are some kinds of anti-aging agents to decrease the thermal-oxidative aging of asphalt, few anti-aging agents were developed based on the volatilization of saturates and aromatics during the construction of asphalt pavement. Finally, the anti-aging agent compositions developed for decreasing the thermal-oxidative aging of asphalt are single, and not compounded according to the thermal properties and contents of saturates and aromatics at the component level.

Based on our previous studies [31], the harmful volatiles of asphalt were mainly produced from aromatics and saturates due to the thermal-oxidative aging reactions during the construction of asphalt pavement, bringing adverse impacts on ecological environment and workers’ health. Therefore, the objective of this study is to first select the corresponding anti-aging agents for aromatics and saturates based on their thermal properties and released volatile constituents, respectively, and then the selected anti-aging agents are compounded according to the contents of aromatics and saturates in asphalt. Thus, a new composite anti-aging agent for asphalt material is developed to improve the thermal stability and reduce the thermal-oxidative aging of asphalt, lowering the release amount of volatiles during the construction of asphalt pavement.

In this study, MH and CaCO_3_ nanoparticles were first selected as anti-aging agents of saturates and aromatics, respectively, improving their thermal stability and reducing the release amount of volatiles. Then, EG was selected as a carrier of MH and CaCO_3_ nanoparticles, and also adsorbed and fixed light gaseous products released from saturates and aromatics in asphalt. After that, EG/MH and EG/CaCO_3_ were added into saturates and aromatics as their anti-aging agents, respectively. The thermal-oxidative aging process of saturates and aromatics was stimulated by the isothermal TG/DSC-FTIR test. Effects of EG/MH and EG/CaCO_3_ on the thermal stability and released volatile constitutes of saturates and aromatics were discussed by TG/DSC-FTIR test results.

Further, the composite anti-aging agent of EG/MH/CaCO_3_ was developed based on inhibitory actions of EG/MH and EG/CaCO_3_ on the thermal-oxidative aging of saturates and aromatics. EG/MH/CaCO_3_ was added into a neat asphalt and the isothermal TG/DSC-FTIR test was used to stimulate the short-term thermal-oxidative aging of asphalt. Finally, the influences of developed composite anti-aging agent of EG/MH/CaCO_3_ on the thermal stability, volatile emission, morphology, and elemental content of asphalt were characterized using isothermal TG/DSC-FTIR and (Environment Scanning Electric Microscope-Energy Disperse Spectroscopy test) ESEM-EDS tests. This study develops an efficient composite anti-aging agent for asphalt materials to reduce the thermal-oxidative aging of asphalt at the component level, improving the durability of asphalt pavement. 

## 2. Materials and Methods

### 2.1. Raw Materials

In this study, the used base asphalt with a 60/80 penetration grade was bought from California Texas Oil Company, USA. SARA fractions were separated from the asphalt binder according to ASTMD4124-09. The contents of saturates, aromatics, resins, and asphaltenes were 18.4%, 40.7%, 30.9%, and 10% by weight, respectively.

EG was selected as an adsorbent to load MH and CaCO_3_ for preparing the composite anti-aging agent of saturates and aromatics, respectively. MH and CaCO_3_ have been surface modified by a silane coupling agent. The expansion rate of EG is 210 mL/g. The grain sizes of MH and CaCO_3_ are 30–50 nm and 40–80 nm, respectively. The purity of MH and CaCO_3_ are both more than 98%. Basic properties of neat asphalt and composite anti-aging agent modified asphalt were summarized in Table 1.

### 2.2. Sample Preparation

#### 2.2.1. Dosage Determination of Anti-Aging Agents 

It is known that EG and nano MH had a good synergistic effect when their content ratio was 5:6 by weight [32]. When the content ratio of EG and nano CaCO_3_ was 5:12 by weight, the mass loss of asphalt during the heating was the smallest [33]. Thus, the mixed ratio of EG to MH was decided as 5:6 to add into saturates, and the mixed ratio of EG to CaCO_3_ was 5:12 to add into aromatics. According to the contents of saturates and aromatics in asphalt, the mixed ratio of EG, MH, and CaCO_3_ in the prepared composite anti-aging agent was 2:1:3. As a result, the added dosages of EG/MH in saturates, EG/CaCO_3_ in aromatics, and EG/MH/CaCO_3_ in asphalt were 10 wt.% according to the previous study [23]. As a powder additive, the influences of composite anti-aging agent on the basic performances of asphalt were considered. Basic properties of asphalt containing a 10 wt.% composite anti-aging agent were tested as provided in Table 1, indicating that performance indexes of composite anti-aging agent modified asphalt met technical standard requirements.

#### 2.2.2. Preparation of Samples Containing Composite Anti-Aging Agents

Firstly, 50 mg EG was dried in a vacuum oven at 60 °C for 5 h. The dried EG was heated in a furnace at 800 °C and expanded for 60 s to obtain the expanded EG with an expansion volume of 210 mL/g. Secondly, MH and CaCO_3_ nanoparticles were doped on EG by a hydrothermal synthesis. Ten mg expanded EG was mixed with 12 mg MH and 24 mg CaCO_3_ in the deionized water, respectively. Then, the two mixed solutions were transferred to two teflon-lined autoclaves. The autoclaves were maintained at 120 °C for 3 h, and then naturally cooled to room temperature. The prepared samples were washed three times using the centrifugation with deionized water and absolute ethanol, as well as dried in an oven at 60 °C to obtain EG/MH and EG/CaCO_3_ [34,35]. Thirty mg expanded EG was mixed with 15 mg MH and 45 mg CaCO_3_ in the deionized water. Then, EG/MH/CaCO_3_ was prepared by repeating the above steps. 

Thirdly, saturates, aromatics, asphalt, EG/MH, EG/CaCO_3_, and EG/MH/CaCO_3_ were placed in a drying oven with a vacuum degree of 93 ± 1 kPa at 105 °C for 1 h. Then, EG/MH, EG/CaCO_3_, and EG/MH/CaCO_3_ were gradually added in saturates aromatics and asphalt at the content of 10 wt.%, respectively, as well as stirred using the shear dispersion machine (FM300 type, FLUKO Equipment Co., Ltd, Shanghai, China) at 1000 rpm for 5 min, followed by a higher stirring speed of 4000 rpm for 20 min. Finally, the hand stirring was used to prevent segregation and remove the air bubbles until the prepared samples were cooled to room temperature. 

### 2.3. Methods

#### 2.3.1. Isothermal TG/DSC-FTIR Test

Based on our previous study [31], the TG/DSC test system (STA 409, Netzsch, Germany) coupled with a FTIR spectrometer (Nicolet IS10, Thermo scientific, Grand Island, NY, USA) was used to evaluate effects of anti-aging agents on thermal-oxidative aging of saturates, aromatics, and asphalt, respectively. Approximately 10 mg of the sample was placed in alumina crucibles of the TG/DSC test system. The heat temperature was raised to 163 °C from room temperature at a heating rate of 40 °C/min and maintained at 163 °C for 4 h based on the Chinese Standard Test Methods of Bitumen and Bituminous Mixture for Highway Engineering [36]. Then, 21% oxygen and 79% nitrogen were input at a flow rate of 60 mL/min. Thus, thermal-oxidative aging tests of saturates, aromatics, and asphalt samples were performed to discuss the changes in mass loss, heat enthalpy, and volatile constituents during the isothermal heating before and after adding anti-aging agents, respectively. The linear baseline method was used to calculate the area of endothermic or exothermic peaks on the DSC curve. The area between the baseline and an endothermic or exothermic peak on the DSC curve was calculated to obtain enthalpy.

At the same time, the released volatiles were imported into the combined FTIR analyzer ((Nicolet IS10, Thermo scientific, Grand Island, NY, USA) by the purge gas at a flow rate of 120 mL/min. The FTIR test results were recorded continuously to identify the released volatile constituents. Before the formal isothermal TG/DSC-FTIR test of each sample, the temperature and balance calibration were performed, and preliminary experiments for repeatability examination under the same experiment conditions were conducted three times. When the results showed that the TG and DSC curves superposed perfectly and the errors were acceptable, the formal experiment of each sample was carried out. Therefore, four samples were prepared to characterize effects of the anti-aging agent on thermal-oxidative aging properties of saturates, aromatics, and asphalt, respectively. 

#### 2.3.2. ESEM–EDS Test

The changes of micromorphology and main element contents of asphalt samples before and after the above simulated isothermal-oxidative aging were characterized by ESEM (Quanta 200 type, FEI, Grand Island, NY, USA) equipped with an EDS. Firstly, asphalt with the sample size of 1 cm × 1 cm × 1 cm was prepared and placed on the clean sample table using a conductive adhesive. Then, the gold powder was sprayed on the asphalt sample and the observation chamber was vacuumized until the pressure reached −3.06 × 10^−3^ Pa. Sample morphologies were immediately observed using ESEM, and the chemical compositions were detected using EDS. The chemical element contents were average values of three random test points on each sample.

## 3. Results and Discussion

### 3.1. Thermal Stability Effect of Anti-Aging Agents on Saturates And Aromatics

To discuss the effects of prepared anti-aging agents of EG/MH and EG/CaCO_3_ on the thermal stability of saturates and aromatics, isothermal TG/DSC-FTIR tests were conducted to simulate the thermal-oxidative aging at 163 °C for 4 h. 

#### 3.1.1. Thermal Stability Effect of EG/MH on Saturates

**Figure 1:**
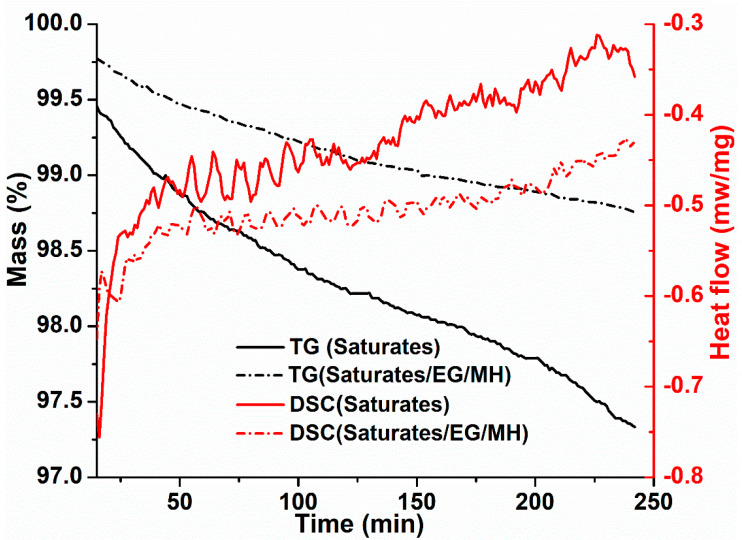
TG and DSC curves of saturates and saturates/expanded graphite (EG)/magnesium hydroxide (MH) during the isothermal-oxidative aging.

TG and DSC curves of saturates and saturates/EG/MH during the isothermal-oxidative aging are shown in Figure 1.

From the TG curves in Figure 1, it is found that the mass of saturates is decreased as the thermal-oxidative aging duration is prolonged before and after adding EG/MH. The decreasing rate of saturates/EG/MH is much smaller than that of saturates. This indicates that the decomposition reaction and the volatilization of light components in saturates are decreased after adding EG/MH during the thermal-oxidative aging of saturates. This is because EG/MH plays the filling and stabilizing roles in saturates, and the porous EG has a strong adsorption performance so that some short-chain hydrocarbon molecules are adsorbed by EG [12]. 

Since the heating temperature is constant, the change in heat flow is mainly due to the temperature difference between saturates and the reference material samples. The DSC curves of saturates and saturates/EG/MH fluctuate continuously at 163 °C for 4 h, and the changing amplitude of saturates is larger than that of saturates/EG/MH. This suggests that different components in saturates participate in the chemical reactions at different heating stages during the isothermal-oxidative aging of saturates. Moreover, the relatively intense and complex endothermic and exothermic reactions occur. This is because saturates are light components in asphalt, and main molecular chains and lateral chains are easily fractured at high temperature to release or adsorb heat during the isothermal-oxidative aging of saturates. 

Additionally, the heat flow values of saturates and saturates/EG/MH show an increasing trend on the whole, but the heat flow values of saturates are higher than those of saturates/EG/MH. This indicates that saturates undergo an endothermic reaction as a whole during the isothermal-oxidative aging at 163 °C for 4 h. It is found that the DSC curves show that the reaction heat is obviously decreased after adding EG/MH in saturates. The reason is that EG adsorbs small hydrocarbon molecules and insulates oxygen and heat, hindering the further thermal-oxidation. MH is loaded on porous EG to fix small molecules, filling and stabilizing saturates [11,23]. The synergetic effects of EG and MH increase the thermal stability of saturates, and the chemical reactions are weakened, leading to the decrease in reaction heat during the isothermal-oxidative aging.

#### 3.1.2. Thermal Stability Effect of EG/CaCO_3_ on Aromatics

**Figure 2:**
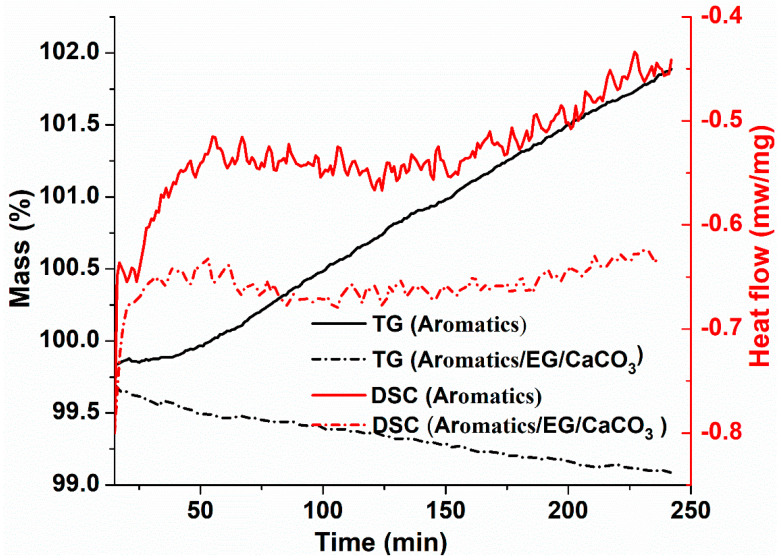
TG and DSC curves of aromatics and aromatics/EG/calcium carbonate (CaCO_3_) during the isothermal-oxidative aging.

Isothermal TG/DSC tests are conducted on aromatics before and after adding EG/CaCO_3_ at 163 °C for 4 h. TG and DSC curves aromatics and aromatics/EG/CaCO_3_ during the isothermal-oxidative aging are shown in Figure 2.

From the TG curves in Figure 2, it is noted that aromatics without the anti-aging agent of EG/CaCO_3_ show a slight increasing trend in mass, while the mass of aromatics after adding EG/CaCO_3_ in aromatics is slightly decreased during the isothermal-oxidative aging. This indicates that the polymerization reaction in aromatics is more than the decomposition reaction before adding EG and CaCO_3_ nanoparticles, producing some macromolecular products. This is because aromatics are transformed to resins due to the oxidative polymerization during the isothermal-oxidative aging, and the lateral chains on aromatic rings are easy to be oxidized and polymerized to form aromatic cycloalkyl [37]. However, the polymerization reaction in aromatics is less than the decomposition reaction after adding EG/CaCO_3_, resulting in the decrease in mass of aromatics during the isothermal-oxidative aging. 

This is mainly due to the fact that some light components are adsorbed and fixed by EG to prevent the generation of macromolecules through further oxidative polymerization reaction with oxygen [38]. However, a small amount of small molecular components is also escaped during the thermal decomposition reaction of aromatics. When the mass of emitted volatile is larger than that of the produced macromolecules by polymerization reaction, the total mass of aromatics is slightly decreased. Meanwhile, the hydrophilic CaCO_3_ nanoparticles loaded on the edge and inner wall of EG uniformly disperses in aromatics to form a cross-linking structure, improving the thermal stability of aromatics, and causing its mass loss to decrease more slowly.

As shown in Figure 2, DSC curves show that the relatively intense and complex endothermic reactions occur in the isothermal-oxidative aging process of aromatics, suggesting that different components in aromatics participate in the chemical reactions at different heating stages during the isothermal-oxidative aging. After adding EG/CaCO_3_, the changing trend of heat flow of aromatics is almost parallel to the abscissa. This suggests that the thermal stability of aromatics is improved, which is owing to the synergistic action between EG and CaCO_3_. The absorption of EG hinders the chain reaction, suppressing the thermal-oxidative aging process of aromatics. Surface effects of CaCO_3_ nanoparticles further stabilize the physical state of aromatics, improving the thermal stability of aromatics. 

### 3.2. Inhibition of Anti-Aging Agents on Volatile Release of Saturates and Aromatics

In order to identify the volatile constituents during the isothermal-oxidative aging, FTIR test results are recorded continuously in the simulated thermal-oxidative aging process. 

#### 3.2.1. Inhibitory Effects of EG/MH on Volatile Release from Saturates

**Figure 3:**
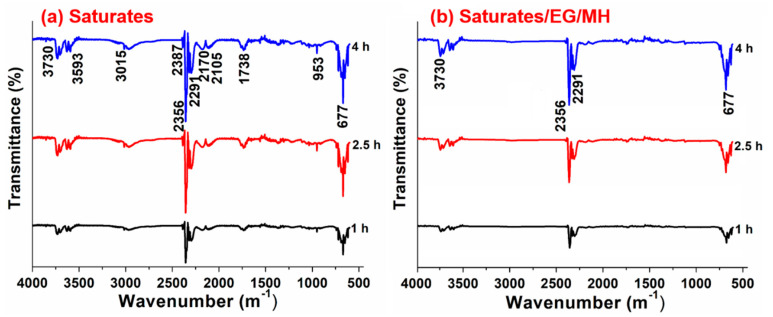
FTIR test results of volatiles released from (**a**) saturates and (**b**) saturates/EG/MH during the isothermal-oxidative aging.

FTIR test results of volatiles released from saturates and saturates/EG/MH during the isothermal- oxidative aging are shown in Figure 3.

Figure 3 shows that the functional group kinds of volatiles in the isothermal-oxidative aging process are reduced after adding EG/MH and the intensities of characteristic peaks after different aging durations are lowered. This implies that EG has been effective in the adsorption and immobilization of light gaseous products, resulting in a decrease in the escape of small short-chain hydrocarbon molecules. The characteristic absorption peak at 3015 cm^−1^ is caused by the stretching vibration of unsaturated hydrocarbon C–H bond. After the addition of EG/MH, the stretching vibration of the unsaturated hydrocarbon C–H bond disappears. These results show that after the addition of EG/MH, the unsaturated C–H bonds in saturates are first oxidized, then the decomposition action occurs. This is because that the MH nanoparticles play the role of size effect and uniformly distributes in saturates, filling and further improving the thermal stability of saturates. Strong characteristic absorption peaks at 2356 and 677 cm^−1^ are due to the antisymmetric stretching vibration and deformation vibration of CO_2_, respectively, indicating that a large amount of CO_2_ is released during the isothermal-oxidative aging of saturates. This is mainly because of the deacidification reaction of carboxyl group (–COOH), the reaction of the hydroxyl group (–OH), and the fracture of aliphatic hydrocarbon side chain, etc. The band at 1738 cm^−1^ is the characteristic absorption peak of stretching vibration of carbonyl group (C=O), suggesting the presence of aldehyde, etc. After adding EG/MH into saturates, the release amount of CO_2_ is significantly reduced, and the characteristic peak of C=O disappears. The out-of-plane bending vibration of olefin C–H at 953 cm^−1^ disappears, indicating that a small amount of unsaturated double bonds in saturates/EG/MH are oxidized so that the thermal stability of saturates is enhanced. This suggests that the aging chain reactions in saturates are slowed down, and EG and MH have a good synergistic inhibitory effect to retard the thermal-oxidative aging of saturates.

#### 3.2.2. Inhibitory Effects of EG/CaCO_3_ on Volatile Release from Aromatics 

**Figure 4:**
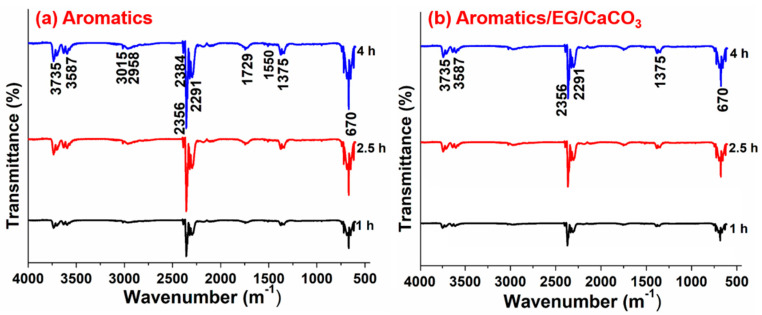
FTIR test results of volatiles released from (**a**) aromatics and (**b**) aromatics/EG/CaCO_3_ during the isothermal-oxidative aging.

Figure 4 shows the FTIR test results of volatiles released from aromatics and aromatics/EG/CaCO_3_ during the thermal-oxidative aging.

It is found from Figure 4 that the main constituents of volatiles released from aromatics during the thermal-oxidative aging are low molecular hydrocarbons, alcohols, and gaseous products such as CO_2_, SO_2_, etc. After adding EG/CaCO_3_ into aromatics, the characteristic peak number of volatile is decreased during the same aging duration, and the intensity of characteristic absorption peak becomes weaker accordingly. This indicates that the addition of EG/CaCO_3_ effectively inhibits the thermal decomposition of aromatics. Especially, the characteristic absorption peaks at 3015 and 2985 cm^−1^ are due to the stretching vibrations of unsaturated hydrocarbon bonds. The unsaturated C–H bonds in volatiles weaken and disappear after adding EG/CaCO_3_. This is due to the uniform dispersion of hydrophilic CaCO_3_ nanoparticles, causing the unsaturated bond in aromatics to decrease in the thermal-oxidative process and improving the thermal stability of aromatics. This is consistent with the above test results of DSC. The bands at 2356 and 670 cm^−1^ are attributed to an antisymmetric stretching vibration and deformation vibration of CO_2_, respectively. After adding EG/CaCO_3_ in aromatics, the release amount of CO_2_ is decreased significantly, and the stretching vibration of unsaturated hydrocarbon bond disappears since EG adsorbs and fixes some small molecules.

The bands at 3587 and 1729 cm^−1^ indicate the presence of –COOH and its derivatives. After adding EG/CaCO_3_ in aromatics, the functional group of C=O disappears, which is also an evidence that the addition of CaCO_3_ increases the thermal stability of aromatics and suppresses the thermal-oxidation of aromatics. The band at 1375 cm^−1^ is caused by the antisymmetric stretching vibration of S=O, indicating the presence of sulfur. After adding EG/CaCO_3_ in aromatics, the peak at 1375 cm^−1^ still exists, suggesting that the addition of EG/CaCO_3_ has no obvious inhibitory effect on the thermal-oxidation of sulfide. In general, the addition of EG/CaCO_3_ inhibits the volatilization of thermal decomposition products with small molecular weights, then further suppresses the thermal-oxidation and decomposition, improving the thermal stability of aromatics to a certain extent.

### 3.3. Improvements of Developed Composite Anti-Aging Agent on Asphalt

Based on the above improvements of EG/MH and EG/CaCO_3_ on the anti-aging properties of saturates and aromatics, respectively, the composite anti-aging agent of EG/MH/CaCO_3_ is developed to improve the anti-aging performance of asphalt. The improvements of developed composite anti-aging agent of EG/MH/CaCO_3_ on asphalt are discussed using TG/DSC-FTIR, revealing the inhibitory mechanism of EG/MH/CaCO_3_ on the thermal-oxidative aging behaviors of asphalt.

#### 3.3.1. Thermal Stability Improvement of EG/MH/CaCO_3_ on Asphalt 

**Figure 5:**
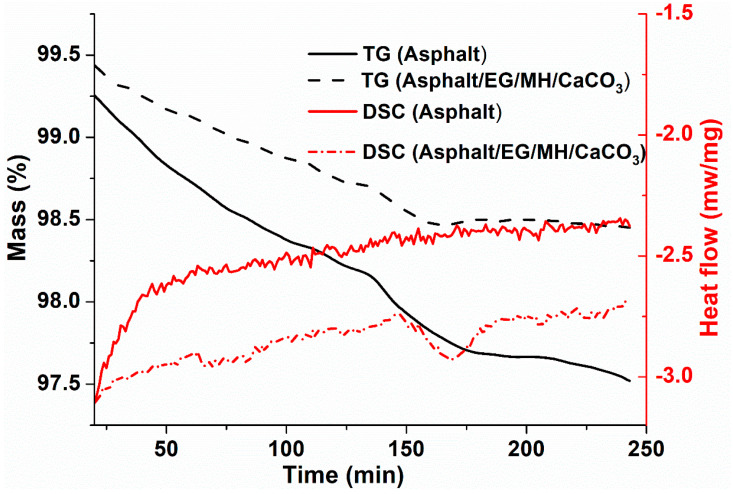
TG and DSC curves of asphalt and asphalt/EG/MH/CaCO_3_ during the isothermal-oxidative aging.

TG and DSC curves of asphalt and asphalt/EG/MH/CaCO_3_ during the isothermal-oxidative aging are shown in Figure 5.

From the TG curves in Figure 5, it is found that the asphalt mass is decreased both before and after adding EG/MH/CaCO_3_ as the isothermal-oxidative aging duration is prolonged. However, the mass loss of asphalt is decreased after adding the composite anti-aging agent of EG/MH/CaCO_3_. This indicates that volatile organic compounds in the process of thermal-oxidative aging of asphalt are reduced after being modified by EG/MH/CaCO_3_. Moreover, from the DSC curves in Figure 5, the heat flow of asphalt/EG/MH/CaCO_3_ increases more slowly than that of asphalt during the isothermal-oxidative aging. This is because EG absorbs partial volatiles, as well as MH and CaCO_3_ nanoparticles loaded on porous EG effectively play the filling roles to improve the thermal stability of asphalt. However, a concave peak indicates that an exothermic reaction appears at around 170 min. This is related to the neutralization reaction between the volatile carboxylic acid and its derivatives with the alkaline MH in EG/MH/CaCO_3_ during the isothermal-oxidative aging of asphalt. 

#### 3.3.2. Inhibitory Effects of EG/MH/CaCO_3_ on Volatile Release of Asphalt

**Figure 6:**
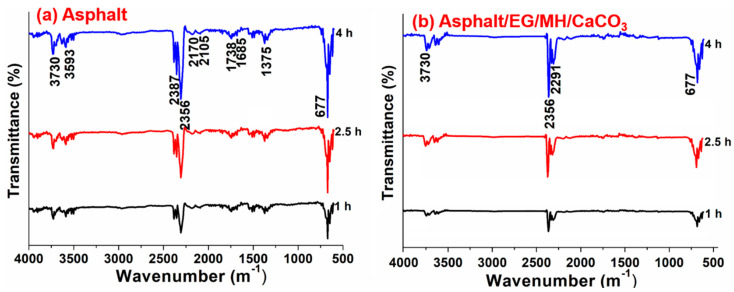
FTIR test results of volatiles released from (**a**) asphalt and (**b**) asphalt/EG/MH/CaCO_3_ during the isothermal-oxidative aging.

To discuss the volatile constituent changes before and after adding EG/MH/CaCO_3_, FTIR tests are conducted and test results are recorded continuously during the isothermal-oxidative aging of asphalt as presented in Figure 6. 

From Figure 6, it is seen that the characteristic peak intensities at 1375, 1685, and 1738 cm^−1^ are decreased until they almost disappear, indicating that S=O and C=O concentrations in volatiles are significantly decreased. Moreover, the bands at 2356 and 670 cm^−1^ are because of the presence of CO_2_, which is decreased after adding EG/MH/CaCO_3_ in asphalt. 

All these suggest that the release amount of volatiles, low molecular hydrocarbons, alcohols, CO_2_ and SO_2_ in the thermal-oxidative aging process is decreased after adding EG/MH/CaCO_3_ in asphalt. The reason is that EG absorbs some small molecules volatiles, the loaded MH and CaCO_3_ nanoparticles play the roles of quantum size effect and fix the small molecules, inhibiting further thermal-oxidation and thermal decomposition. This improves the thermal stability of asphalt, and reduces the release amount of volatiles. The developed composite anti-aging agent of EG/MH/CaCO_3_ shows an obvious inhibitory effect on the isothermal-oxidative aging of asphalt.

#### 3.3.3. Morphology and Elemental Changes of Asphalt and Asphalt/EG/MH/CaCO_3_

**Figure 7:**
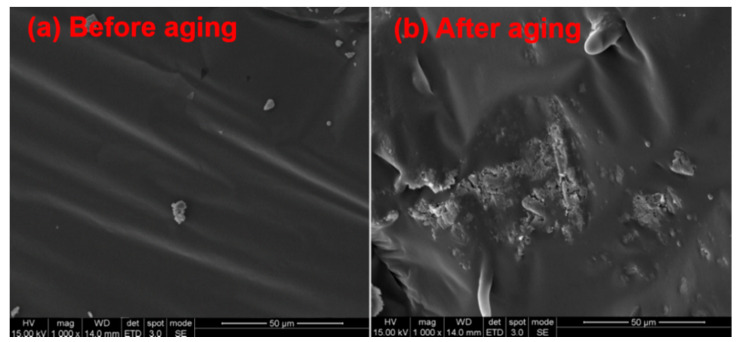
SEM images of asphalt (**a**) before and (**b**) after the isothermal-oxidative aging.

**Figure 8:**
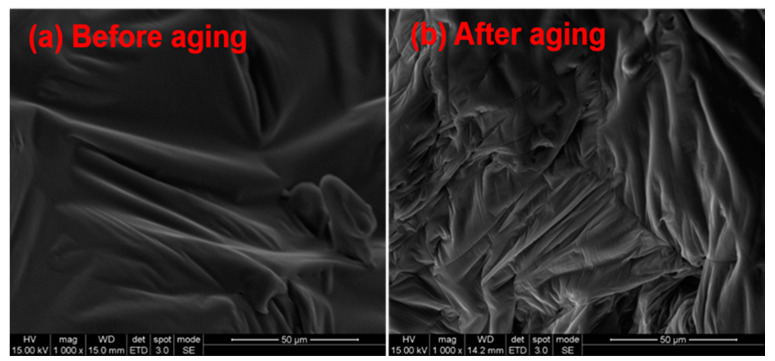
SEM images of asphalt/EG/MH/CaCO_3_ (**a**) before and (**b**) after the isothermal-oxidative aging.

To further understand the anti-aging effects of EG/MH/CaCO_3_ on the thermal-oxidative aging and the release of volatiles, microscopic morphology changes of asphalt before and after adding EG/MH/CaCO_3_ are discussed. Figure 7 and Figure 8 show the SEM images of asphalt and asphalt/EG/MH/CaCO_3_ before and after the isothermal-oxidative aging, respectively. Elemental changes of asphalt and asphalt/EG/MH/CaCO_3_ before and after the isothermal-oxidative are summarized in Table 2. 

As presented in Figure 7a, the surface of asphalt before the isothermal-oxidative aging is relatively regular, flat, and dense, and a small amount of solid particles are found, which reflects the sol-gel structure of asphalt. After the isothermal-oxidative aging, an obvious fold appears on the asphalt surface which becomes dry, rough, and uneven. On one hand, this is mainly due to the partial volatilization of light organic compounds during the simulated isothermal-oxidative aging at 163 °C for 4 h. Saturates and aromatics are decreased, but resins and asphaltenes are increased on the surface of asphalt [37]. 

On the other hand, combined with the test results of TG and DSC, it is inferred that the polymerization reaction occurs during the continuous heating to produce long carbon chains and macromolecular compositions, so that the asphalt surface becomes dry, rough, and uneven. In addition, Table 2 shows that the contents of C and O elements are increased, while the content of S element is decreased on the surface of asphalt. This is mainly because carbon oxides are generated by thermal-oxidative polymerization [29]. A small amount of SO_2_ generated by sulfur oxidation is released from the surface of asphalt, resulting in the decrease in S element.

It is seen from Figure 8a that asphalt/EG/MH/CaCO_3_ is still a sol-gel structure. The surface is relatively flat, dense, smooth, and moist, indicating that the composite anti-aging agent of EG/MH/CaCO_3_ is evenly distributed in asphalt. As shown in Figure 8b, after the isothermal-oxidative aging, the dense striped folds are found on the surface of asphalt. Moreover, no obvious holes and dye are found on the surface of asphalt. This indicates that the loss of light oil components of saturates and aromatics, as well as macromolecular products generated by polymerization are decreased in the process of isothermal-oxidative aging of asphalt after adding EG/MH/CaCO_3_. This is also due to the fact that the added EG adsorbs and fixes partial small molecules, reducing the volatilization of light organic compounds. Moreover, the SEM images prove that the addition of MH produces an oxide film on the surface of asphalt and forms a physical barrier with the carbon layer generated by the expansion of EG [16].

Moreover, MH nanoparticles fill the holes where some released light organic compounds leave and CaCO_3_ nanoparticles increase the thermal stability of asphalt, which reduces the progress of the thermal-oxidative reaction of asphalt to a certain extent [18]. Simultaneously, the light organic compounds in asphalt and the free radicals generated by thermal-oxidization can be partially inserted between EG layers or adsorbed on the surface of EG. This prevents the chain reaction during the isothermal-oxidative aging of asphalt so that the aging process is suppressed [39]. Furthermore, from Table 2, EDS test results show that Ca and Mg elements are detected on the surface of asphalt after adding EG/MH/CaCO_3_, which proves the filling effects of MH and CaCO_3_ nanoparticles.

## 4. Conclusions

In this study, thermal-oxidation characteristics and volatile constituents of asphalt modified by composite anti-aging agents are investigated both at the SARA and the whole level by the isothermal TG/DSC-FTIR test. Based on the comprehensive test results and discussion, main conclusions are summarized as follows:

(1) After adding EG/MH, the exothermic reaction of saturates weakens, and the thermal-oxidative aging reaction slows down. MH plays a filling role and improves the thermal stability of saturates. At high temperature, the light components of saturates and the free radicals generated by the thermal oxidation are inserted between the EG layers or adsorbed on the surface of EG, which suppresses the thermal oxidation of saturates.
(2) The reaction heat in the thermal-oxidative aging process of aromatics decreases after adding EG/CaCO_3_. CaCO_3_ leads to thermal hysteresis and improves the thermal stability of aromatics. The light organic compounds and polycyclic aromatic hydrocarbons generated by the thermal-oxidative aging of aromatics are adsorbed and fixed by EG, hindering the chain reaction, and suppressing the process of thermal-oxidative aging of aromatics.
(3) MH can not only improve the thermal stability of saturates, but also reduce carboxylic acid and its derivatives through reacting with –COOH groups, which hinders the further oxidation of saturates. CaCO_3_ exerts its size effect, and plays the role of immobilizing small molecules, and improves the thermal stability of asphalt to some extent because of the synergistic reaction with EG.
(4) The added proportions of EG, MH, and CaCO_3_ in the developed composite anti-aging agent of EG/MH/CaCO_3_ are 2:1:3 by weight. EG plays a synergistic role with MH and CaCO_3_ nanoparticles, which prevents the chain reactions, inhibiting the thermal-oxidative aging of asphalt.
(5) The developed EG/MH/CaCO_3_ plays a satisfactory effect on inhibiting the thermal-oxidative aging of asphalt and reduces the release of volatile organic compounds in the thermal-oxidative aging process of asphalt. This study develops a new composite anti-aging agent for asphalt, and provides an insight into the improvements of EG/MH/CaCO_3_ on reducing thermal aging properties of asphalt.

## Figures and Tables

**Table 1:** Basic properties of neat asphalt and composite anti-aging agent modified asphalt.

Properties		Standards	Neat Asphalt	Modified Asphalt
Penetration at 25 °C (dmm)		ASTM D5-06	69	65
Ductility at 15 °C (cm)		ASTM D113-07	146	135
Softening point (R&B) (°C)		ASTM D36-06	48	57
Viscosity at 60 °C (Pa·s)		ASTM D4402-06	298	330
Flash point (°C)		ASTM D92-05	320	325
Solubility (%)		ASTM D2042-09	99.9	98.8
Density (g/cm^3^)		ASTM D70-09	1.033	1.036
Tests after RTFOT	Mass change (%)	ASTM D2872-04	0.28	0.19
	Ductility at 15°C (cm)	ASTM D113-07	121	108
	Penetration ratio at 25°C (%)	ASTM D5-06	75	70

**Table 2:** Elemental content changes of asphalt and asphalt/EG/MH/CaCO_3_ before and after the isothermal-oxidative aging.

Element (wt.%)	Asphalt		Asphalt/EG/MH/CaCO_3_	
	Before Aging	After Aging	Before Aging	After Aging
C	86.1	87.7	89.0	91.6
O	5.7	6.6	3.8	4.6
S	5.3	1.8	4.8	4.5
Mg	-	-	4.7	2.7
Ca	-	-	0.6	0.5
